# Nrf2 Is Crucial to Graft Survival in a Rodent Model of Heart Transplantation

**DOI:** 10.1155/2013/919313

**Published:** 2013-02-28

**Authors:** Wei Wu, Quan Qiu, Huihui Wang, Samantha A. Whitman, Deyu Fang, Fangru Lian, Donna D. Zhang

**Affiliations:** ^1^Department of Pharmacology and Toxicology, The University of Arizona, Tucson, AZ 85721, USA; ^2^Department of Cardiothoracic Surgery, Southwest Hospital, Third Military Medical University, Chongqing 400038, China; ^3^Department of Pathology, School of Medicine, Northwestern University, Chicago, IL 60611, USA; ^4^Department of Pathology, The University of Arizona, Tucson, AZ 85724, USA

## Abstract

Currently, the sole treatment option for patients with heart failure is transplantation. The battle of prolonging graft survival and modulating innate and adaptive immune responses is still being waged in the clinic and in research labs. The transcription factor Nrf2 controls major cell survival pathways and is central to moderating inflammation and immune responses. In this study the effect of Nrf2 levels in host recipient C57BL/6 mice on Balb/c allogeneic graft survival was examined. Importantly, Nrf2^−/−^ recipient mice could not support the graft for longer than 7.5 days on average, whereas activation of Nrf2 by sulforaphane in Nrf2^+/+^ hosts prolonged graft survival to 13 days. Several immune cells in the spleen of recipient mice were unchanged; however, CD11b^+^ macrophages were significantly increased in Nrf2^−/−^ mice. In addition, IL-17 mRNA levels were elevated in grafts transplanted into Nrf2^−/−^ mice. Although Nrf2 appears to play a crucial role in graft survival, the exact mechanism is yet to be fully understood.

## 1. Introduction

Heart transplantation is the only restorative technique for end-stage heart failure, and allograft rejection remains a significant barrier to successful transplantation. The immune response to an allograft is an ongoing interchange between the innate and adaptive immune systems, which if left unchecked will ultimately lead to rejection of the transplanted organ. In addition, implanted grafts are recognized as “nonself” by the recipient immune system due to discordant major histocompatibility complex (MHC); what is more, other immunogenic signals such as tissue damage resulting from organ procurement and ischemia/reperfusion damage can also promote host immune responses and graft rejection. 

The transcription factor nuclear factor (erythroid-derived 2)-like 2 (NFE2L2) or “Nrf2” is a master regulator of the cellular antioxidant response. Nrf2 promotes cell survival by binding the antioxidant response element (ARE) in the promoter of downstream target genes that promote detoxification of xenobiotics and scavenging of reactive oxygen species (ROS). The benefit of Nrf2 activation has been demonstrated in various pathological states such as diabetes, chemoprevention, and cardiovascular and neurological diseases [1–5]. 

The role of Nrf2 in mediating cellular inflammation and immunity is rapidly becoming established. Activation of Nrf2 appears to antagonize inflammatory pathways such as TGF*β*1 and NF-*κ*B [6, 7]. Nrf2's influence on survival of transplants (i.e., liver or cardiac stem cells) has been investigated from the side of the donated tissue [8–10]. However, Nrf2 genotype and/or modulation on the side of the recipient host have not yet been investigated. The purpose of the present study was to determine how Nrf2 in recipient hosts affects graft survival in an allogeneic mouse model of heart transplantation.

## 2. Methods

### 2.1. Animals and Surgery

**Figure 1:**
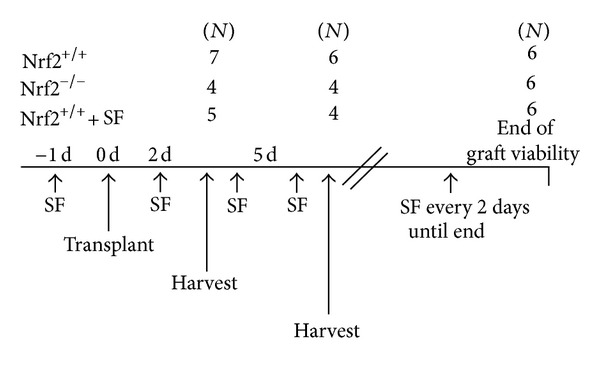
Study design diagram. A schematic of the study design, time points, and *N*-size for each group is provided. Briefly, donor hearts and recipient spleens were harvested at 2 days and 5 days following transplant, and donor hearts were harvested at the final day of graft survival for analysis. For the Nrf2^+/+^ group treated with sulforaphane (SF), animals were injected 1 day prior to transplant and then every 48 h until tissues were harvested.

Nrf2^+/+^ (WT) and Nrf2^−/−^ (KO) C57BL/6 mice (described previously [11]) aged 8–12 weeks were used as recipient mice for heart transplant studies. Six–eight-week-old Balb/c mice were purchased from Jackson Labs and used as donors for heart transplant studies. All animals received water and food *ad libitum*. Nrf2 WT mice were left untreated or given sulforaphane (SF) (WT + SF) by i.p. injection (12.5 mg/kg body weight) one day prior to transplant and every 48 h after transplant until animals were sacrificed. KO mice were left untreated, or a smaller subset also received SF treatment exactly as described previously to assess the specificity of SF acting via the Nrf2 pathway (data not shown). Recipient animals were sacrificed, and organs were harvested for analysis at day 2 and day 5 after transplant. Additional recipient animals were monitored daily to assess viability of the donor heart by monitoring beating under the skin in the neck. These recipient mice were sacrificed, and organs were harvested for analysis once the graft was no longer viable. A diagram of the study design and animal groups (including *N*-size) is provided in Figure 1. 

The mouse model of cervical heterotopic cardiac allograft was performed using the Heron cuff technique and has been described previously in mice [12]. In brief, the right cervical common carotid artery and external jugular vein in recipient C57BL/6 mice were isolated for anastomosis. Clamps were placed at the most caudal end of the carotid artery and external jugular vein, whereas the cranial ends were ligated and severed. A Teflon “cuff” was placed over the caudal carotid artery and jugular vein, and the Balb/c donor aorta and pulmonary artery were connected to the cannula of the recipient cervical common carotid artery and external jugular vein and were ligated and fixed. Blood supply was immediately resumed by releasing the caudal clamps, and the cervical incision was closed if the transplanted heart was beating well without obvious bleeding. The University of Arizona Institutional Animal Care and Use Committee approved all protocols for animal handling and surgery. 

### 2.2. Histology

 Transplanted donor hearts were formalin fixed and paraffin embedded, and sections were cut at ∼4 *μ*m thickness for hematoxylin and eosin (H&E) staining and immunohistochemistry (IHC) analysis. Hearts stained with H&E were evaluated for general histology and rejection according to the International Society for Heart and Lung Transplantation [13]. Briefly, heart sections from day 2 and day 5 following transplantation were evaluated by a trained pathologist and assessed for the grade of rejection defined as follows: 1R = mild, focal perivascular and/or interstitial infiltrate without myocyte damage; 2R = medium, multifocal infiltrate with myocyte damage; 3R = severe, diffuse polymorphous infiltrate with extensive myocyte damage and/or edema and/or hemorrhage and/or vasculitis; or “Quilty” = inflammation from the outside-in, that is, starting from the epicardium and encroaching on the myocardium, possibly due to infection rather than rejection.

### 2.3. Flow Cytometry Analysis

Single-cell suspensions of spleen were obtained by mincing the organs through a cell strainer dish and stained according to standard procedures. If not mentioned, all the antibodies were purchased from BioLegend (San Diego, USA). Flow cytometry was performed on a BD FACSCanto or Accuri C6 flow cytometer (BD Biosciences, San Jose, USA). Cell surface staining of CD3, CD4, CD8, B220, NK, NKT, macrophages, and DCs was performed following standard protocols; total splenocytes were stimulated with PMA (50 ng/mL)/Ionomycin (1 *μ*g/mL) (*in vitro *with monensin) for 4 h; then intracellular staining of FoxP3, IFN-*γ*, IL17, and IL4 was done using the eBioscience staining buffer. All the data were analyzed using FlowJo software (FlowJo, Ashland, USA) excluding cell doublets. 

### 2.4. RNA Isolation and qPCR

Total RNA was isolated from donor heart tissues using Trizol. RNA quality was assessed using a NanoDrop (Wilmington, DE) where the 260/280 ratios were obtained. Samples with a ratio of 1.7–2.1 were utilized for downstream gene analysis. Approximately 1 *μ*g total RNA from each sample was reverse-transcribed using M-MLV (Promega, Madison, WI), oligo-DT, random primers, and dNTPs from Roche (Indianapolis, IN). cDNA was then diluted *∼*1 : 25 in nuclease free water to be used for qPCR. All primers used were intron spanning, and gene expression levels were analyzed using TaqMan chemistry with primers and probes designed in Roche's Universal Probe Library Design Center. All experiments were conducted on a LightCycler 480 (Roche). Relative gene expression was calculated using the 2^−ΔΔCT^ method [14], and studies were designed according to the MIQE guidelines [15]. For all housekeeping and target genes assessed, standard curves were evaluated on 10-fold serial dilutions of control cDNA to determine reaction efficiency. Where efficiencies between housekeeping and target genes differed >5%, efficiencies could be accounted for in the LightCycler 480 software during analysis of relative gene expression. A complete list of target genes and primers is provided in Table 1. Specificity of primer pairs was evaluated through the use of both no-RT and water template control samples. Only primers that did not amplify more than one product or any product in the no-RT and water controls were used for final experiments. 

### 2.5. Statistical Analysis

One-way ANOVA using the Tukey-Kramer post hoc analysis for differences between means assessed statistical differences between animal groups and time points for survival times and flow cytometry. Data from qPCR was analyzed using relative comparison from the Roche LightCycler software, which is based on the 2^−ΔΔCT^ method. Efficiencies of each primer set were determined by standard curve analysis and considered by the software algorithm for determining relative expression over a housekeeping gene, ornithine decarboxylase (*Odc*).

## 3. Results

### 3.1. Activation of Nrf2 Prolongs Graft Survival of Heart Transplants in Mice

**Figure 2:**
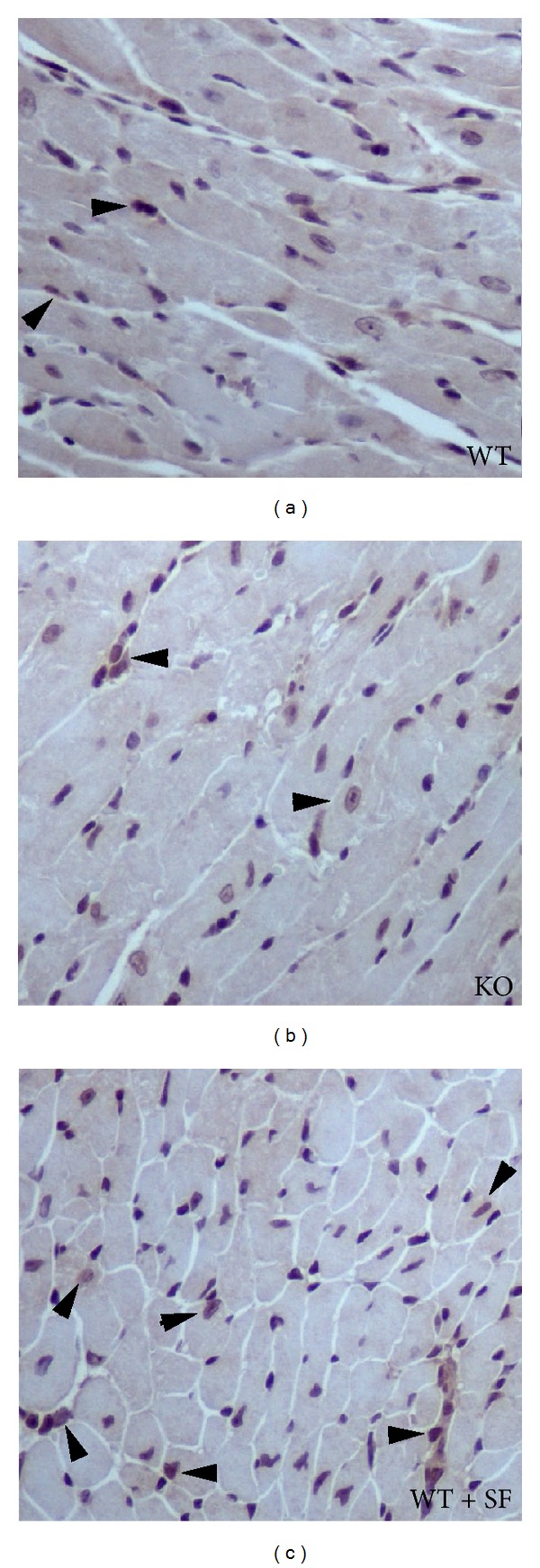
Nrf2 IHC staining in donor Balb/c hearts 2 days following transplant. Formalin-fixed and paraffin-embedded heart from donor Balb/c mice were analyzed for Nrf2 expression two days after heart transplant. Nrf2 levels are moderate as shown by minimal nuclear staining in the C57BL/6 recipient Nrf2 WT (a) and KO (b) groups. Treatment of C57BL/6 recipient Nrf2 WT with SF (c) increases nuclear expression of Nrf2 in donor. Arrowheads denote positive nuclear staining.

 To examine the role of  Nrf2 in heart transplant graft survival, hearts from Balb/c mice were transplanted into C57BL/6 Nrf2^+/+^ (WT) with or without sulforaphane (WT + SF) treatment or Nrf2^−/−^ (KO) mice.  Table 2  illustrates that loss of Nrf2 function in mice results in a significantly shorter survival time of the Balb/c graft when compared to their wild-type counterparts, implying that Nrf2 has a protective role in graft survival. Notably, treatment of recipient Nrf2^+/+^ mice with the Nrf2 activator SF significantly extended the survival of the graft to nearly 13 days.  Figure 2 provides a representative illustration of Nrf2 levels in grafts 2 days after transplantation. Note the minimal Nrf2 expression in a few nuclei of the Balb/c donor hearts transplanted into Nrf2 WT (Figure 2(a)) and KO (Figure 2(b)) mice, whereas Nrf2 expression is increased in grafts where Nrf2 WT recipient mice received SF treatment (Figure 2(c)). It is important to note that treatment with SF in the Nrf2^−/−^ mice showed no effects on overall survival time of the graft (data not shown), indicating that SF treatment protects the heart graft from rejection largely through Nrf2 activation. Therefore, activation of Nrf2 has a great therapeutic potential to prolong graft survival in transplantation. 

### 3.2. Treatment of Recipient Mice with Sulforaphane Delays Transplant Rejection

**Figure 3:**
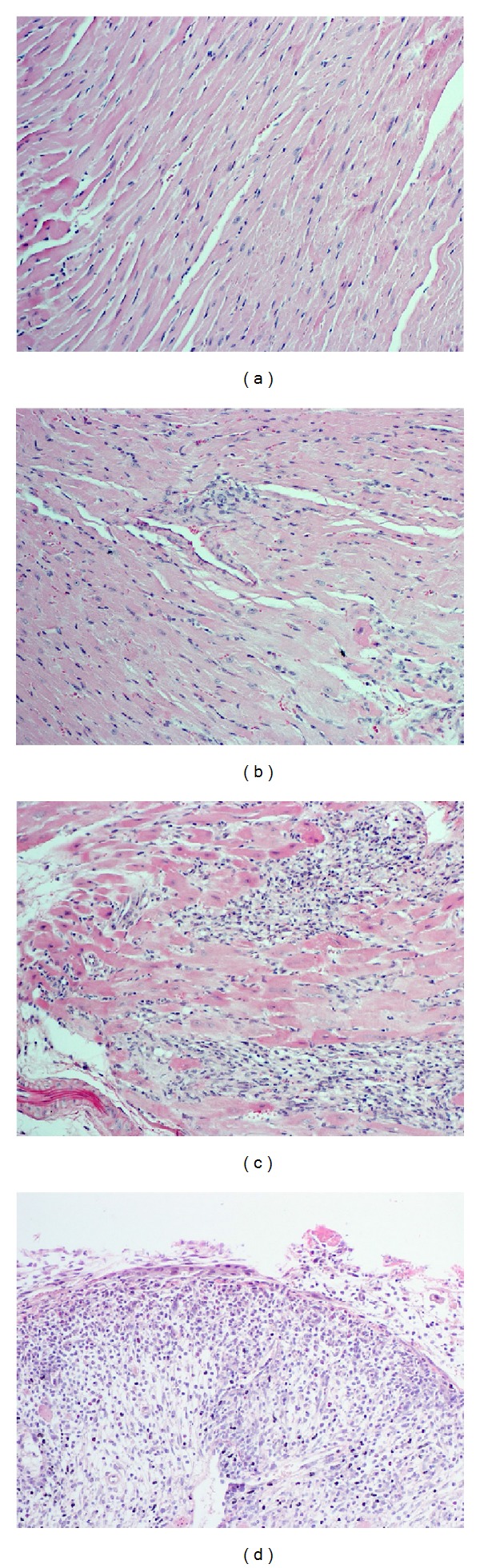
Representative histology from donor Balb/c hearts. Hearts isolated at 2 and 5 days following transplant were analyzed by histology for rejection grading. Representative images display no rejection (a), Grade 1 rejection (b), and Grade 2 rejection (c) or “Quilty” phenomenon (d). Images were taken at 20x.

 Donated Balb/c hearts were analyzed at 2 and 5 days following transplantation for histology and rejection classification. A summary of the analysis and complete data presentation is provided in Table 3, and representative images of the pathological histology used to categorize each animal are displayed in Figure 3. In summary, Nrf2^+/+^ and ^−/−^ animals showed some low-grade rejection (see Figure 3(b) for representative image of Grade 1R) at 2 days after transplant as similar levels of lymphocyte infiltration were observed. Remarkably, Nrf2^+/+^ animals pretreated with SF showed no histological signs of rejection 2 days after transplant (see Figure 3(a) for representative image), suggesting that activation of Nrf2 can delay transplant rejection. However, all animals showed significant signs of graft rejection 5 days following transplant (see Figure 3(c) for representative image of Grade 2R), with Nrf2^+/+^ animals all displaying signs of “Quilty,” a phenomenon thought to be independent of rejection, showing infiltrate originating from the epicardium and infiltrating inward (see Figure 3(d) for representative image of “Quilty”). These data suggest that activation of Nrf2 with SF is able to delay graft rejection and infiltrates of immune cells for at least the first 2 days after transplant, possibly contributing to the significant increase in overall graft survival observed in these animals.

### 3.3. Nrf2 Influences IL-17 Production in the Spleen of Recipients

**Figure 4:**
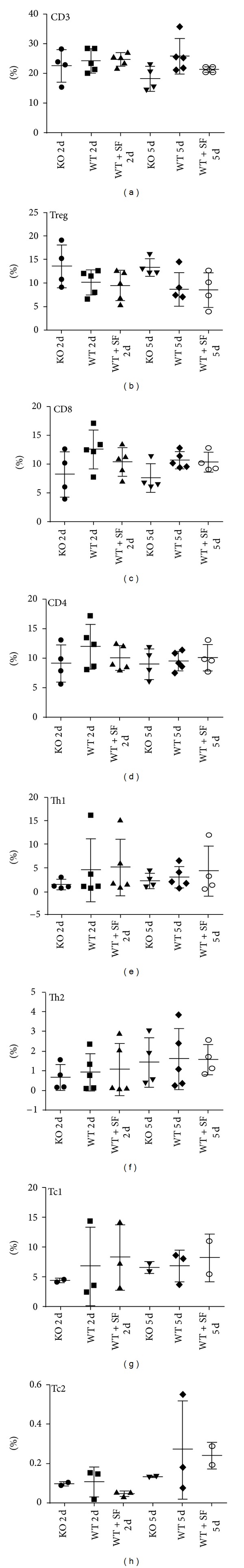
T-cell populations are generally unchanged in spleens from transplant recipients. Flow cytometry analysis on spleens isolated from recipient mice (Nrf2 WT, KO, or WT + SF) at either day 2 (2 d) or day 5 (5 d) following transplantation. No significant differences were found in T-cell populations including total (CD3^+^)(a), Tregs (b), CD8^+^ (c), CD4^+^ (d), Th1^+^ (e), Th2^+^ (f), Tc1^+^ (g), or Tc2^+^ (h). *N*-size ranged from 4 to 7 animals per group, per time point.

**Figure 5:**
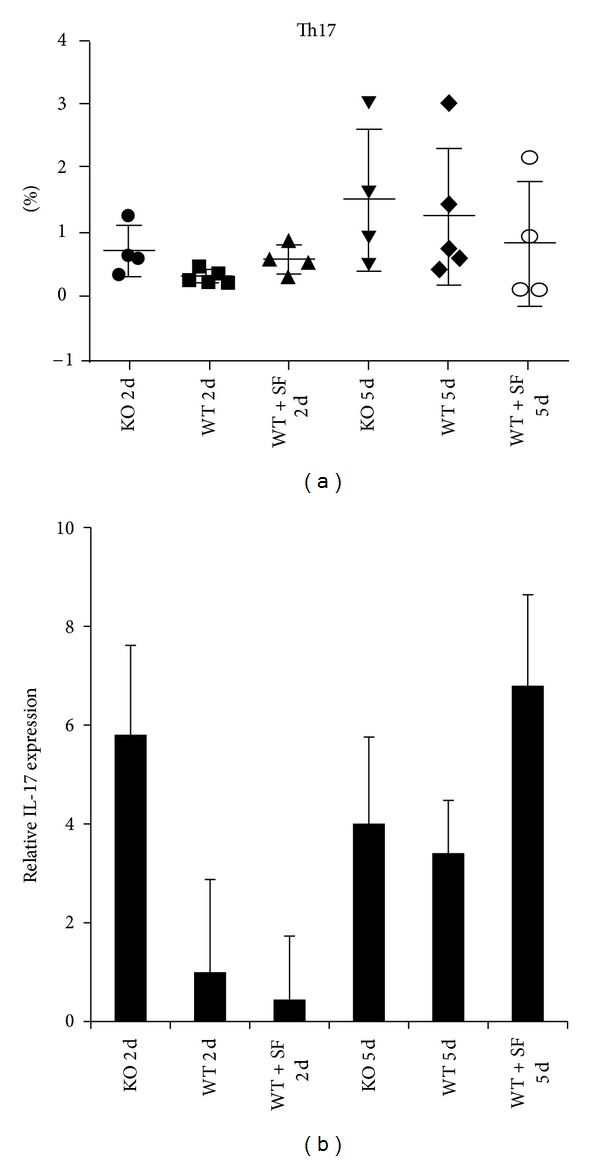
Th17 and IL-17 trend higher in spleen and grafts, respectively, from Nrf2 KO recipients. Flow cytometry analysis of spleens from recipient mice (Nrf2 WT, KO, or WT + SF) at day 2 (2 d) after transplantation shows a trend toward increased Th17 cells in the KO group (a). This is supported by notably higher expression of IL-17 mRNA in grafts isolated from Nrf2 KO recipients at 2 days (b), with no differences between groups at 5 days.

T cells are predominating players for acute graft rejection (for review, see [16]). To investigate a potential mechanism mediating rejection in the Nrf2 KO mice, we analyzed the T-cell immune responses in the spleens of recipient mice. There were no remarkable differences in the numbers of total T cells (CD3^+^), CD4^+^, CD8^+^, Th1, Th2, Tc1, or Tc2 in the spleens of recipients (Figure 4). However, mRNA levels of the Th17 cytokine and IL-17 were markedly higher in the donated hearts transplanted to KO recipients at 2 days following transplant (Figure 5). Together with the notion that loss of Nrf2 function facilitates heart graft rejection (Table 2), the elevated IL-17 production in the graft likely contributes to the rejection. Consistent with the histological analysis, at 5 days after transplant IL-17 mRNA levels in the donated hearts are elevated in all recipient groups regardless of genotype or treatment, implying that other factors exist in the graft-specific immune response due to the altered Nrf2 function.

### 3.4. Nrf2 KO Mice Generate a Significantly Greater Macrophage Population in Response to Transplant

**Figure 6:**
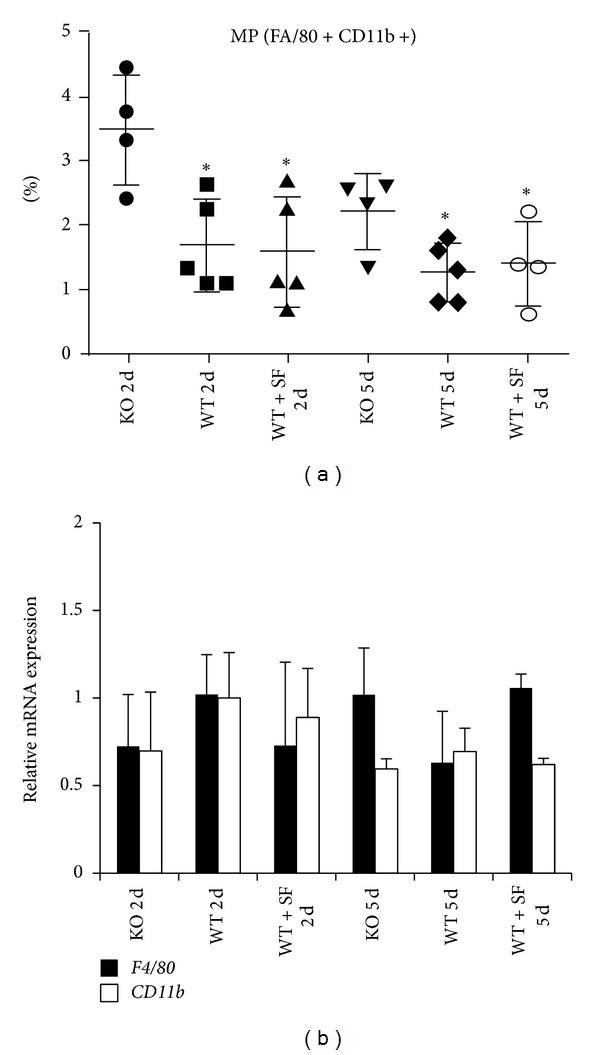
CD11b^+^ macrophages increase significantly in spleens of Nrf2 KO recipients. (a) Flow cytometry analysis on spleens isolated from recipient mice (Nrf2 WT, KO, or WT + SF) at either day 2 (2 d) or day 5 (5 d) after transplantation shows a significant increase in CD11b^+^ cells in the KO spleens. (b) illustrates relative mRNA levels of *CD11b* (solid bars) and *F4/80* (striped bars) in donated Balb/c hearts at 2 and 5 days after transplant. No notable change above 2-fold was observed between the Nrf2 recipient groups.

**Figure 7:**
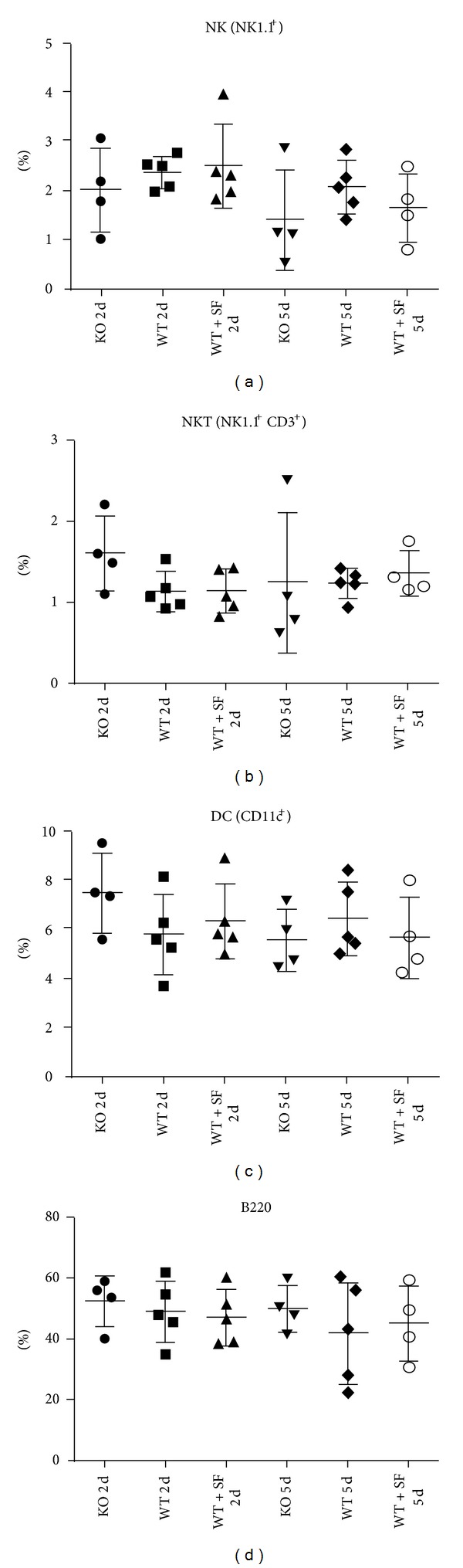
No alterations in non-T-cell immune populations regardless of Nrf2 genotype. Flow cytometry analysis on spleens isolated from recipient mice (Nrf2 WT, KO, or WT + SF) at either day 2 (2 d) or day 5 (5 d) following transplantation did not reveal other differences in non-T-cell immune cells such as NK (a), NKT (b), dendritic cells (c), or B cells (d).

Evaluation of spleens from recipient C57BL/6 mice for non-T-cell immune cells revealed a significant elevation in CD11b^+^F4/80^+^ macrophages in KO mice (Figure 6(a)), whereas other non-T-cell populations such as NK, DC, and B cells are unchanged regardless of genotype, treatment, or time point after transplantation (Figure 7). These data imply a possible link of elevated macrophage population in the spleens of the KO recipient mice to heart graft rejection. To assess whether this additional macrophage population in the KO animals correlated with increased macrophage infiltration of the graft, we evaluated CD11b expression by IHC, as well as CD11b and F4/80 mRNA expression in the grafts from each animal group. Analysis by IHC for CD11b in formalin-fixed, paraffin-embedded grafts yielded inconclusive results due to minimal positive staining within the graft (data not shown). Additionally, evaluation of mRNA levels of CD11b and F4/80 did not show any notable changes in expression (less than 2-fold) between groups when data were normalized to the Nrf2 WT day 2 group (Figure 6(b)).

## 4. Discussion

Currently heart transplantation is the only option for end-stage heart failure disease, and prolonging graft survival is still the subject of intense research. In this study the effects of Nrf2 on host defense and allogeneic graft survival were examined. Our results support that Nrf2 is required to maintain graft survival and that activation of the Nrf2 pathway could prove to be beneficial in extending the life of the transplanted heart. 

The role of Nrf2 in the innate and adaptive immune systems continues to be uncovered. Recently, Nrf2 has been implicated in altering maturation of dendritic cells (DCs) [17, 18]. In addition, activation of Nrf2 in older mice upregulates Th1 immunity by maintaining DC redox equilibrium [19]. Although we did not directly assess DC maturity or costimulatory responses in the current study, no alterations in total DC population were observed in the spleens of Nrf2 WT, KO, or WT + SF treated mice. What is more, activation of Nrf2 by the common food preservative, *tert*-butylhydroquinone (tBHQ), appears to skew CD4^+^ T cells toward Th2 differentiation; however, there were no observable differences in CD4^+^ or Th2^+^ T cells in any of our treatment groups. In the current study we utilized a well-known Nrf2 activator, the isothiocyanate sulforaphane. One possibility is that the mechanism of action of either or both of these activators could have off-target effects that influence T-cell differentiation through a non-Nrf2-mediated pathway. 

Nrf2 KO mice have been shown to suffer from splenomegaly and spleen cell death and inflammation in older age, also resulting in hemolytic anemia caused by increased sequestration of IgG-bound erythrocytes in the spleen [20]. Although loss of Nrf2 has been implicated in other autoimmune disorders [21], the anemia in the study by Lee et al. was instead damage induced from increased oxidative stress. It is hard to determine whether an underlying immune-mediated anemia in the KO mice in this study could have affected the systemic immune response to graft rejection; however, given the young age of the mice in the present study, it is unlikely. 

In the case of bacterial infection and sepsis, Nrf2 has been shown to contribute to mobilizing the immune response and counteract the oxidative stress in monocytes [22, 23]. In fact Nrf2 is critical at modulating an “appropriate” level of inflammatory response so as not to be damaging to the host organs and cells. Loss of Nrf2 dramatically impacts survival during experimental sepsis due to deregulated inflammation and activation of Nrf2 can improve survival [24]. Some evidence of this was seen in the present study where CD11b^+^ macrophages were significantly elevated in the spleen of Nrf2 KO at 2 days after heart transplantation, indicating a disproportionate innate response in the early days after transplant. However, follow-up analysis by IHC and qPCR could not conclusively provide evidence of increased macrophage infiltration in the Nrf2 KO recipient animals. Nrf2 WT mice when given SF did not display a significantly lower CD11b^+^ count. One possibility is that Nrf2 activation by SF was able to minimize the innate response to early graft rejection thereby extending the overall graft survival by ∼5 and ∼3 days over KO and WT mice, respectively. Taken together, the increased macrophage number in the KO spleens may not result in increased infiltration into the graft; however, increased macrophages in the peripheral lymphoid organs could result in production of additional factors such as cytokines, ROS activity, and stress responses, which lead to the earlier rejection.

An additional avenue worth pursuing in the future of this work is the specific scavenging of ROS that is mediated by the Nrf2 pathway. Ischemia-reperfusion injury is a common issue with organ transplantation and has been shown previously to dictate the rejection response over that of recipient immunity [25]. One explanation of poor transplant tolerance in Nrf2 KO mice could be due to their lack of ability to scavenge ROS. Ischemia-reperfusion injury inherently generates high levels of ROS that establish an environment of inflammation and infiltrating leukocytes. Treatment of recipient Nrf2 WT mice with SF could decrease ROS production and assist in minimizing inflammation as well as altering the chemokine environment in the graft to delay intrusion of recipient immune effectors.

Interestingly, there were only subtle differences between T-cell and non-T-cell populations in the recipient spleens of mice in this study. Although CD11b^+^ macrophages were the only cell population that reached statistical significance, Th17 cells did trend to being higher in Nrf2 KO mice, which was supported by the overwhelming presence of IL-17 mRNA in grafts taken 2 days after transplant from KO mice. Conversely, IL-17 mRNA was notably reduced in grafts harvested 2 days after transplant from WT mice treated with SF. These data support that though there may not be differences in T-cell differentiation or total population, perhaps trafficking of immune cells is somehow different between Nrf2 WT and KO genotypes. 

## 5. Conclusions 

The work presented here displays the importance of Nrf2 in graft survival in an allogeneic mouse model of heart transplant. These data provide an initial insight into the therapeutic benefit of Nrf2 activation during transplant. Yet more work needs to be done to prove the hypotheses laid forth in this study, as well as to fully interrogate how Nrf2 (and its activation) is able to prolong graft survival upon heart transplant. Though the mechanism is critically important, the implications of using Nrf2 activators in a clinical setting to improve allogeneic graft survival are established by our current work. 

## Figures and Tables

**Table 1:** Primers used in qPCR experiments.

Target gene	Forward 5′-3′	Reverse 5′-3′
*Odc *	GCCAGTAACGGAGTCCAGAT	ATCATCAGTGGCAATCCGTA
*IL-17 *	TGTGAAGGTCAACCTCAAAGTC	GAGGGATATCTATCAGGGTCTTCA
*CD11b *	AGCCCCACACTAGCATCAA	TCCATGTCCACAGAGCAAAG
*F4/80 *	GGAGGAGACATCCACTCTGG	TGATGACTTTGCTTTCGATGTC

**Table 2:** Overall survival of Balb/c donor hearts.

Recipient genotype/treatment	WT	KO	WT + SF
Survival time (days)	9.63	7.25	16
	10.63	7.33	11.6
	9.83	6.42	11.77
	9.38	6.5	12.3
	9	7.2	12.33
	9.75	7.5	12.66

Time in days (Ave ± SD)	9.07 ± 0.54	7.03 ± 0.46*	12.76 ± 1.63^∗,#^

Raw data from graft survival along with the average and standard deviation is provided for each group. *P < 0.05 compared to WT; ^#^ *P* < 0.05 compared to KO.

**Table 3:** Sulforaphane pretreatment delays graft rejection.

	WT day 2 (grade *N*/total *N*)	KO day 2 (grade *N*/total *N*)	WT + SF day 2 (grade *N*/total *N*)	WT day 5 (grade *N*/total *N*)	KO day 5 (grade *N*/total *N*)	WT + SF day 5 (grade *N*/total *N*)
No rejection	1/5	2/3	5/5			
Grade 1R	1/5	1/3		1/4	1/4	
Grade 2R	3/5			3/4	3/4	3/3
Quilty				4/4		

*N* = 3–5 animals for each group. Data is displayed as “*N*”—meeting histological criteria/total analyzed “*N*.” Heart sections were formalin fixed and paraffin embedded prior to being stained with H&E for histology analysis. Definition of terms: 1R = mild, focal perivascular and/or interstitial infiltrate without myocyte damage, 2R = medium, multifocal infiltrate with myocyte damage, “Quilty”: inflammation from the outside-in, that is, starting from the epicardium, possibly due to infection rather than rejection.
